# Impact of the Zero Maternal Death by Hemorrhage Strategy on health professionals’ self-perceived knowledge in managing postpartum hemorrhage

**DOI:** 10.61622/rbgo/2025rbgo25

**Published:** 2025-05-04

**Authors:** Gilson Geraldo de Oliveira, Mariana Larissa Oliveira dos Santos, Gabriel Costa Osanan, George Dantas de Azevedo, Reginaldo Antônio de Oliveira Freitas

**Affiliations:** 1 Universidade Federal do Rio Grande do Norte Natal RN Brazil Universidade Federal do Rio Grande do Norte, Natal, RN, Brazil.; 2 Universidae Federal de Minas Gerais Belo Horizonte MG Brazil Universidae Federal de Minas Gerais, Belo Horizonte, MG, Brazil.; 3 Instituto de Ensino e Pesquisa Alberto Santos Dumont Macaíba RN Brazil Instituto de Ensino e Pesquisa Alberto Santos Dumont, Macaíba, RN, Brazil.

**Keywords:** Medical education, Healthcare professionals, Health strategies, Postpartum hemorrhage, Maternal mortality

## Abstract

**Objective:**

This study aimed to evaluate the results of the Obstetric Hemorrhage Prevention and Management Course – Zero Maternal Death by Hemorrhage Strategy (0MMxH) among healthcare professionals before and after participation.

**Methods:**

A quasi-experimental design was employed, assessing the educational intervention in a convenience sample of healthcare professionals who had participated in the 0MMxH at least one year prior. Participants completed a retrospective pre-post questionnaire sent via email, focusing on self-perceived knowledge levels and the adoption of best practices in postpartum hemorrhage (PPH) management.

**Results:**

Out of 129 professionals who completed the 0MMxH training, 85 (65.9%) responded to the questionnaire. The percentages of respondents reporting no or low knowledge before and after the course were: shock index (52.8% to 0%, before and after, respectively), blood loss estimation (35.2% to 1.1%), care sequence for PPH (44.6% to 0%), rational use of crystalloids (37.5% to 1.1%), non-pneumatic anti-shock garment (83.5% to 3.4%), and damage control surgery (74.1% to 8.1%). These results indicate a significant improvement in self-perceived knowledge. After the course, the highest adoption rates of best practices were for shock index (83.5%), blood loss estimation (67.1%), and use of warm crystalloids (58.8%). However, gaps remained regarding non-pharmacological interventions for PPH management.

**Conclusion:**

Participants reported improved knowledge on most topics covered by the 0MMxH. The program was recognized as a crucial factor in adopting effective PPH management practices, underscoring the importance of training in enhancing obstetric care.

## Introduction

Postpartum hemorrhage (PPH) can be defined as a 500 mL loss of blood after vaginal delivery or 1000 mL after a Cesarean section; or any loss of blood accompanied by signs or symptoms of hemodynamic instability, regardless of route of delivery, within 24 hours after birth. PPH is the leading cause of maternal mortality worldwide, with most deaths considered avoidable and occurring in low- and middle-income countries. The causes of PPH can be summarized by the four "T's": tone (uterine atony), trauma (uterine laceration or rupture), tissue (retained placenta or clots), and thrombin (coagulation disorders).^(1–7)^

Effective PPH management requires a coordinated multidisciplinary approach, involving clear communication, accurate assessment of blood loss, continuous monitoring of maternal vital signs and symptoms, fluid resuscitation, and immediate identification and control of the bleeding source, all carried out simultaneously.^(3)^ In this context, the connection between knowledge and prompt intervention directly impacts the decline in complications and prevention of PPH-related maternal mortality.^(2,4,6)^ Thus, it is essential to adequately train healthcare professionals for accurate clinical assessment and timely action.^(7)^

Zero Maternal Deaths from Hemorrhage (0MMxH) in the Americas is a collaborative project led by the Pan American Health Organization/World Health Organization (PAHO/WHO) and its Latin American Center for Perinatology, Women and Reproductive Health (CLAP/SMR). The initiative aims to strengthen health systems, eliminate barriers to healthcare access, train professionals to manage obstetric hemorrhages, and ensure the availability of necessary medical supplies and equipment to treat severe forms of PPH. It also aims to educate women to fully recognize their rights and preferences. Additionally, the initiative seeks to mobilize governments, civil society, and communities.^(4,8)^

The project has been successfully implemented in the Americas and the Caribbean since 2014. In 2018, the 0MMxH Strategy was adapted to the Brazilian context and national needs via the creation of new educational materials for theoretical classes, skills training stations, simulations, and the appropriate methodology for training Brazilian professionals.^(4,8)^

Self-perception of knowledge refers to how individuals assess their own knowledge and skills regarding specific topics. This concept is widely used in studies within the fields of health and education, as it provides valuable insights into how people perceive their learning outcomes. Although self-perception may either underestimate or overestimate the real competencies of medical students and physicians, its evaluation remains important, understanding that a high self-perception in a certain activity would be related to a greater willingness to perform that activity.^(9,10)^

As such, the aim of this study was to assess the impact of the Zero Maternal Death by Hemorrhage Strategy (0MMxH) on self-perceived knowledge and the adoption of good PPH practices among professionals.

## Methods

This is a quasi-experimental study with a pre- and post-intervention design. A convenience sample of healthcare professionals participating in the 0MMxH training was evaluated for self-perceived knowledge and the adoption of best practices both before and after the intervention. There was no random assignment of participants, and all eligible healthcare professionals who attended the training were invited to participate in the study. All participants provided written informed consent, as well as consenting to image and voice use.

The 0MMxH strategy was implemented from August to September 2021 in the state of Rio Grande do Norte, Brazil. Data were collected one year after strategy implementation. This temporal approach makes it easier to assess the effectiveness of healthcare professional training and its practical application in emergency obstetric care.

A retrospective-pre-post questionnaire was used to analyze self-reported changes in knowledge level and adoption of good PPH management practices. The retrospective-pre-post questionnaire is an effective technique for measuring self-reported changes in aspects such as knowledge, awareness, skills, confidence, attitudes, and behaviors. Its advantages are shorter data collection time, higher adherence, and less sensitivity bias in pre-tests and response change, which can lead to over or underestimations in pre-intervention assessments.^(11–13)^

Knowledge-related data included 16 theoretical contents covered in the course and grouped into three categories: (I) initial assessment of the patient with PPH (PPH diagnosis, methodology used to estimate blood loss, Golden Hour concept, shock index and administration of oxytocin as PPH prevention strategy); (II) overall measures and pharmacological treatment (care sequence for patients with PPH, uterine atony treatment with oxytocin, methylergometrine and misoprostol, use of tranexamic acid as adjuvant in PPH treatment, rational use of crystalloids in hemostatic resuscitation of patients with PPH, early blood transfusion indication based on clinical criteria, lethal triad in PPH: acidosis, hypothermia and coagulopathy); and (III) non-pharmacological treatment (use of non-pneumatic antishock garment as a transient method to control PPH, use of intrauterine balloon tamponade in uterine atony, hemostatic sutures, PPH management by placental acretism and damage control surgery in PPH.

Participants were asked to self-assess their knowledge before and after the course using a five-point Likert scale, as follows: none (had no knowledge of the subject); low (little knowledge of the subject); neither high nor low (some knowledge of the subject and much to learn); high (sufficient knowledge of the subject); very high (extensive knowledge of the subject, capable of teaching others).

Data on adoption of good practices investigated 12 clinical management practices for patients with PPH that are covered in the skills training and practical simulation activities of 0MMxH. These practices include: revision of the perineum, vagina and episiotomy, use of misoprostol, placental examination after delivery, uterine massage, placenta removal by controlled cord traction, bimanual uterine compression, use of tranexamic acid as adjuvant in pharmacological PPH treatment, routine administration of oxytocin as PPH prevention strategy, early blood transfusion based on clinical criteria, use of warm crystalloids for volume replacement, estimate postpartum blood loss by any method and use of shock index as a clinical parameter of PPH. The study investigated which practices health professionals already adopted even before participating in the training and which they only started to adopt after participating in 0MMxH.

The questionnaire was emailed to participants and filled out in digital format. Subsequent data analysis was conducted using Excel, version 2017, and the free statistical software R, version 4.2.0. Statistical analysis was conducted using the Wilcoxon signed-rank test for paired samples and the Chi-square test for categorical variables to compare the measurements before and after training. A p-value < 0.05 was considered statistically significant.

The study was approved by the Research Ethics Committee, in accordance with ethical and legal guidelines, under *Certificado de Apresentação de Apreciação Ética* 50696921.4.0000.5292 (protocol 4.948.749).

## Results

A total of 129 healthcare professionals involved in obstetric emergency care and who completed the 0MMxH training were invited to participate, with 85 (65.9%) responding to the questionnaire. The sample consisted of 70 women (82.4%) and 15 men (17.6%) and included obstetricians (n=64, 75.3%), anesthesiologists (n=2, 2.4%), and nurses (n=19, 22.4%). Analysis of the responses obtained revealed a significant difference in the self-perceived knowledge level of the participants before and after 0MMxH for the 16 theoretical contents analyzed, as presented in tables 1, 2, and 3.

**Table 1:** Distribution of participants in terms of their knowledge level about the topics related to the initial assessment of patients with PPH, during pre- and post-intervention – category I

Initial assessment	Phase	N n(%)	L n(%)	+/- n(%)	H n(%)	VH n(%)	p-value*
PPH diagnosis	Pre	-	9(10.5)	39(45.9)	32(37.6)	5(5.9)	<0.001
	Post	-	1(1.2)	4(4.7)	33(38.8)	4(55.3)	
Methodology used to estimate blood loss in PPH	Pre	6(7.0%)	24(28.2)	40(47.0)	15(17.6)	-	0.001
	Post	-	1(1.1)	3 (3.5)	40(47.0)	41(48.2)	
Golden Hour concept in PPH	Pre	7(8.2%)	26(30.5)	25(29.4)	22(25.8)	5(5.8)	0.002
	Post	-	-	4(4.7)	34(40.0)	47(55.2)	
Shock index in PPH	Pre	22(25.8%)	23(27.0)	24(28.2)	15(17.6)	1(1.1)	0.048
	Post	-	-	5 (5.8)	35(41.1)	45(52.9)	
PPH prevention with oxytocin 10 IU IM injection	Pre	5 (5.8%)	13(15.2)	16(18.8)	23(27.0)	28(32.9)	0.014
	Post	-	-	1(1.1)	19(22.3)	65(76.4)	

* Wilcoxon t-test (5% significance level);

N: None; L: Low; +/-: Neither high, nor low; H: High; VH: Very high

**Table 2:** Distribution of participants in terms of their knowledge level about the topics related to overall measures and pharmacological treatment of patients with PPH, during pre- and post-intervention – category II

Overall measures and pharmacological treatment	Phase	N n(%)	L n(%)	+/- n(%)	H n(%)	VH n(%)	p-value*
Care sequence for patients with PPH	Pre	16(18.8)	22(25.8)	27(31.7)	20(23.5)	-	0.003
	Post	-	-	6(7.0)	41(48.2)	38(44.7)	
Uterine atony treatment including oxytocin, methylergometrine and misoprostol	Pre	1 (1.1)	10(11.7)	19(22.3)	28(32.9)	27(31.7)	< 0.001
	Post	-	-	3(3.5)	25(29.4)	57(67.0)	
Use of tranexamic acid as adjuvant in PPH treatment	Pre	10(11.7)	12(14.1)	20(23.5)	25(29.4)	18(21.1)	0.001
	Post	1(1.1)	-	5(5.8)	28(32.9)	51(60.0)	
Rational use of crystalloids in hemostatic resuscitation of patients with PPH, avoiding a 3:1 ratio	Pre	6(7.0)	26(30.5)	31(36.4)	17(20.0)	5(5.8)	< 0.001
	Post	-	1 (1.1)	7(8.2)	37(43.5)	40(47.0)	
Early blood transfusion indication based on clinical criteria	Pre	6(7.0)	22(25.8%)	30(35.2)	22(25.8)	5(5.8)	< 0.001
	Post	-	1(1.1)	8(9.4)	37(43.5)	39(45.8%)	
Lethal triad in PPH: acidosis, hypothermia and coagulopathy	Pre	8(9.4)	19(22.3)	28(32.9)	20(23.5)	10(11.7)	< 0.001
	Post	-	-	9(10.5)	43(50.5)	33(38.8)	

* Wilcoxon t-test (5% significance level);

N: None; L: Low; +/-: Neither high, nor low; H: High; VH: Very high

**Table 3:** Distribution of participants in terms of their knowledge level about the topics related to non-pharmacological treatment of patients with PPH, during pre- and post-intervention – category III

Non-pharmacological treatment	Phase	N n(%)	L n(%)	+/- n(%)	H n(%)	VH n(%)	p-value*
Use of non-pneumatic antishock garment as a transient method to control PPH (n=85)	Pre	46(54.1)	25(29.4)	11(12.9)	2(2.3)	1(1.1)	< 0.001
	Post	2(2.3)	1(1.1)	6(7.0)	43(50.5)	33(38.8)	
Use of intrauterine balloon tamponade in uterine atony (n=85)	Pre	25(29.4)	28(32.9)	18(21.1)	12(14.1)	2(2.3)	0.024
	Post	2(2.3)	-	8(9.4)	39(45.8)	36(42.3)	
Hemostatic sutures (n=85)	Pre	27(31.7)	17(20.0)	26(30.5)	11(12.9)	4(4.7)	< 0.001
	Post	1(1.1)	4(4.7)	11(12.9)	43(50.5)	26(30.5)	
PPH management by placental acretism (n=85)	Pre	12(14.1)	20(23.5)	27(31.7)	23(27.0)	3(3.5)	0.001
	Post	-	1(1.1)	8(9.4)	41(48.2)	35(41.1)	
Damage control surgery in PPHP (pelvic packing using the vacuum dressing technique) (n=85)	Pre	34(40.0)	29(34.1)	18(21.1)	4 (4.7)	-	< 0.001
	Post	1(1.1)	6(7.0)	14(16.4)	47(55.2)	17(20.0)	

* Wilcoxon t-test (5% significance level);

N: None; L: Low; +/-: Neither high, nor low; H: High; VH: Very high

Before the course, the highest percentages of respondents, within each category, who self-perceived at the combined levels of no and low knowledge, were observed for the following contents: shock index (52.8%) and methods to estimate blood loss in PPH (35.2%) in category I; care sequence for patients with PPH (44.6%) and rational use of crystalloids in hemostatic resuscitation for patients with PPH (37.5%) in category II; and use of a non-pneumatic anti-shock garment (NASG) as a transient method for PPH control (83.5%) and damage control surgery for PPH (74.1%) in category III. One year after participating in 0MMxH, the response profile for no and low knowledge levels on these specific contents changed to shock index (0%) and methods to estimate blood loss in PPH (1.1%) in category I; care sequence for patients with PPH (0%) and rational use of crystalloids in hemostatic resuscitation for patients with PPH (1.1%) in category II; and use of NASG as a transient method for PPH control (3.4%) and damage control surgery for PPH (8.1%) in category III.

Before the course, the highest percentages of responses at the high and very high knowledge levels, combined and by category, were observed for the following contents: PPH prevention with the intramuscular use of oxytocin 10 IU (59.9%) in category I; pharmacological treatment of PPH (64.6%) in category II; and PPH management due to placental accreta (30.5%) in category III. After the course, self-perception regarding the highest percentages of respondents at the high and very high knowledge levels, combined and by category, were PPH prevention with the intramuscular use of oxytocin 10 IU (98.7%) in category I; pharmacological treatment of PPH (96.4%) in category II; and use of NASG (89.3%) and PPH management due to placental accreta (89.3%) in category III. The lowest percentage of responses at the high and very high levels was observed for damage control surgery for PPH (75.2%) in category III. With respect to the use of good clinical practices for PPH management, the following strategies were most commonly reported as already being utilized prior to participation in 0MMxH: perineum, vagina, and episiotomy revision (92.9%), use of misoprostol in the pharmacological treatment of PPH (91.8%), placenta examination after delivery (87.1%), and uterine massage (87.1%) (Table 4). Regarding the adoption of good clinical PPH management practices, the largest increases in usage by participants, one year after participating in 0MMxH, were observed for the following clinical practices: shock index as a clinical parameter (16.5% had already adopted the practice before participating in 0MMxH, and 83.5% began using it only after participating), estimation of postpartum blood loss (32.9% and 67.1%, pre and post, respectively), use of warm crystalloids for volume replacement (41.2% and 58.8%), and early blood transfusion based on clinical criteria (43.5% and 56.5%) (Table 4).

**Table 4:** Distribution of participants in terms of the use of practices aimed at the clinical management of patients with PPH, before and after the workshop

Clinical PPH management practices	Reports that they already adopted the practice before participating in 0MMxH n(%)	Reports that they only started adopting the practice after participating in 0MMxH n(%)	n(%)
Revision of the perineum, vagina and episiotomy	79(92.9)	6(7.1)	85(100)
Use of misoprostol in the treatment of PPH	78(91.8)	7(8.2)	85(100)
Placental examination after delivery	74(87.1)	11(12.9)	85(100)
Uterine massage	74(87.1)	11(12.9)	85(100)
Placenta removal by controlled cord traction	68(80.0)	17(20)	85(100)
Bimanual uterine compression	56(65.9)	29(34.1)	85(100)
Use of tranexamic acid as adjuvant in pharmacological PPH treatment	54(63.5)	31(36.5)	85(100)
Routine IM administration of oxytocin as PPH prevention strategy	53(62.4)	32(37.6)	85(100)
Early blood transfusion based on clinical criteria	37(43.5)	48(56.5)	85(100)
Use of warm crystalloids for volume replacement	35(41.2)	50(58.8)	85(100)
Estimated postpartum blood loss (by any method)	28(32.9)	57(67.1)	85(100)
Shock index as clinical parameter of PPH	14(16.5)	71(83.5)	85(100)

## Discussion

Preventable maternal mortality related to the quality of obstetric care remains a serious public health issue. These deaths are not randomly distributed among women and reveal the inequities of the societies where they occur, since they are concentrated in developing countries, mainly affecting black women, those with lower income, and little schooling. Brazil has seen progress in recent decades, especially with respect to population access to basic health care. However, indicators of preventable maternal mortality demonstrate that the strategies used have been insufficient.^(14)^

Addressing maternal mortality due to postpartum hemorrhage requires a strategic, collective, and ongoing effort to ensure the structuring of services and professional qualification for effective obstetric care, particularly to ensure prevention, timely diagnosis, and appropriate management of these cases. In this respect, both continuous and permanent health education are essential to reduce maternal mortality from direct and preventable causes such as postpartum hemorrhage.

The decline in preventable maternal deaths will always be the most sensitive and effective indicator of the impact of all these measures, even though the multiplicity of determinants involved limits the direct cause-effect correlations, individually, for each of the actions undertaken in the efforts to reduce maternal mortality. However, studying the transformative potential of initiatives such as the Zero Maternal Deaths from Hemorrhage Strategy, from the andragogical perspective and in terms of changes in the practices of participants, is also crucial in assessing its effectiveness, based on meaningful learning and the possibility of transforming daily professional practices.

The success of learning in Health Sciences is related to adequate means of presenting and discussing content with information that produces voluntary adaptations or behavioral modifications; that is, the use of appropriate strategies may favor the assimilation of knowledge, development of skills, and incorporation of values, thereby allowing for changes in health habits or promoting the learning of professionals in these areas, whether in professional training or permanent health education.^(15)^

According to Knowles et al.,^(16)^ andragogy, which is the art and science of leading adults to learning, is structured from six propositions that clearly define the differences between children and adults in the quality of learners. The authors start from the premise that as people mature, they undergo radical transformations, that is, they become independent and responsible for their decisions; they direct their own lives and learning interests; accumulate experiences that will support and substantiate their learning; their interests concentrate on developing the skills they use in performing their social roles and profession; they expect immediate practical application of what they learn, decreasing their interest in knowledge that will be useful in a distant future, and the motivations that drive them are internal, becoming more intense than their external counterparts.^(16)^ It becomes essential for effective learning to consider these propositions.

Ideally, health education should include assessment stages of participants in terms of reaction, learning, behaviors, and results, including the aims and learning gaps that were or were not achieved by the educational strategy. This dynamic process should foster continuous improvement and development cycles, capable of qualifying care and ensuring appropriate responses to the health needs of the target populations.

The results obtained evoke important discussions about which factors favor or limit positive changes in the practice of health professions. Well-established and validated good practices, supported by scientific evidence as being useful and beneficial for managing PPH cases,^(1–7)^ were not yet adopted by most study participants, healthcare professionals working in reference services for obstetric care, before participating in 0MMxH. This was observed for early and clinically based blood transfusions (56.5%), use of warm crystalloids for volume replacement (58.8%), estimated blood loss (67.1%), and shock index as a clinical parameter in PPH (83.5%). It is noteworthy that the main component of active management of the third stage of labor, routine intramuscular administration of oxytocin 10 IU,^(1–7)^ as one of the most widely used strategies for preventing PPH, was not adopted by 37.6% of these professionals. It is crucial to promote broad discussion about work contexts, professional roles, and qualifications that influence knowing scientific evidence and resisting its concrete application in professional practice. These contexts deserve attention during the planning, execution, and assessment phases of health education measures, as factors capable of directly influencing the effectiveness of educational strategies.

The responses of healthcare professionals regarding self-perceived knowledge one year after participating in the 0MMxH Strategy attest to the success of the course in learning content previously unknown to the vast majority of participants. The use of a non-pneumatic anti-shock garment as a transient method for PPH control deserves to be highlighted as a good example, given that 83.5% of respondents reported having no or low previous knowledge of the topic, which changed to 3.4% of respondents one year after participating in the course.

Overall, the category contents related to non-pharmacological treatment of PPH continued to exhibit the highest percentages of respondents with self-perceived no or low knowledge levels on the topics covered in 0MMxH. The literature also reports greater use of pharmacological methods in hospital services as conduct options for managing PPH, at the expense of non-pharmacological methods.^(17)^ This finding should be emphasized in future editions of the strategy so that educational activities and practical skills training stations that use these contents can achieve even better results. After participating in 0MMxH, PPH damage control surgery for PPH through pelvic packing by vacuum dressing was recognized as both the content with the highest percentage of professionals with no and low knowledge (8.1%) and lowest percentage of high and very high knowledge levels (75.2%). This aspect also deserves special attention during practical skills training for pelvic packing, since with specific indications, the intervention is usually performed on severely ill patients, without satisfactory hemostasis obtained by primary methods.^(18)^ In addition to the correct determination of clinical indication, mastering the technique may result in the ideal moment for its application. Alwy Al-Beity et al.^(19)^ studied the impact of training on clinical follow-up and found a decline in near-miss events associated with PPH. Trained professionals were able to identify PPH at an earlier stage, allowing intervention before evolving to a more severe and potentially fatal condition.^(19)^

PPH is the leading cause of maternal mortality worldwide, making it essential for healthcare teams to prevent, diagnose, and institute non-surgical management within the "golden hour." The simultaneous implementation of multiple guidelines for appropriate therapeutic management of PPH justifies the presence of a well-organized workflow in healthcare units. To reduce the risks and morbidity/mortality associated with PPH, it is essential to implement risk stratification in healthcare services, identifying early risk factors, and optimizing prenatal, delivery, and postpartum care. Systematic use of prophylactic oxytocin, active management of the third stage of labor, and efficient methods for estimating blood loss, along with diagnostic and therapeutic adequacy, should be offered uniformly by healthcare teams. The availability of intrauterine balloon tamponade, non-pneumatic anti-shock garments, blood components, and professionals’ knowledge and skills for their correct use complement the healthcare needs for appropriate non-surgical management of PPH. Finally, valuing women's lives, organizing local healthcare systems, and instituting programs aimed at preventing maternal mortality by improving healthcare professionals’ skills and eliminating access barriers are the major challenges in reducing PHH-related morbidity/mortality.^(1)^

The use of self-perception as a measure in this study represents a limitation, as it may not accurately reflect actual competency or long-term changes in practice.^(9,10,20)^ This limitation is further highlighted by de Melo et al.,^(20)^ who conducted a qualitative study assessing the long-term application of acquired knowledge and skills following PPH simulation training. These authors emphasize that a rigorous analysis of knowledge transfer and real-life PPH management presents significant methodological challenges, including the unpredictability of PPH occurrences and the difficulty of implementing strategies to document these situations in daily clinical practice.^(20)^

We acknowledge that the use of a pre-post test design introduces potential biases that may influence the results. These biases could affect the accuracy of the self-reported data and the observed improvements in knowledge and practices. Therefore, future research employing a more rigorous methodological approach, including control groups and extended follow-up periods, is necessary to more effectively evaluate the sustained impact of the 0MMxH strategy. Additionally, future studies should focus on evaluating maternal health indicators, such as the incidence of near-miss events and maternal mortality rates, to provide a more objective measure of the intervention's effectiveness.

The findings of this study underscore the need for permanent health education measures as powerful tools in addressing preventable maternal mortality causes. There is an indisputable need to maintain scientific updates and clinical skills training for professionals working on the frontlines of obstetric emergencies. This responsibility must be shared across several dimensions, from personal commitment through active knowledge-seeking, to leadership within work teams and managers of healthcare services, reaching different healthcare system management levels, whether public or private.

## Conclusion

Healthcare professionals acknowledged an improvement in their knowledge on most topics covered by the 0MMxH Strategy. Among these topics, those related to the non-pharmacological treatment of PPH were identified as the greatest challenges and needs in professional training. There was a clear alignment between the main knowledge gaps identified by the participants and the significant improvements observed in the adoption of effective clinical management practices for patients with PPH. Participants recognized the 0MMxH strategy as a key factor in facilitating the adoption of best practices for PPH management, highlighting the importance of training in enhancing obstetric care.
